# Variance components associated with long-echo-time MR spectroscopic imaging in human brain at 1.5T and 3T

**DOI:** 10.1371/journal.pone.0189872

**Published:** 2017-12-29

**Authors:** Michael J. Thrippleton, Jehill P. Parikh, Scott I. K. Semple, Bridget A. Harris, Peter J. D. Andrews, Joanna M. Wardlaw, Ian Marshall

**Affiliations:** 1 Centre for Clinical Brain Sciences, University of Edinburgh, Edinburgh, United Kingdom; 2 Clinical Research Imaging Centre, University of Edinburgh, Edinburgh, United Kingdom; 3 Critical Care Medicine, University of Edinburgh, Edinburgh, United Kingdom; Linköping University, SWEDEN

## Abstract

**Object:**

Magnetic resonance spectroscopic imaging (MRSI) is increasingly used in medicine and clinical research. Previous reliability studies have used small samples and focussed on limited aspects of variability; information regarding 1.5T versus 3T performance is lacking. The aim of the present work was to measure the inter-session, intra-session, inter-subject, within-brain and residual variance components using both 1.5T and 3T MR scanners.

**Materials and methods:**

Eleven healthy volunteers were invited for MRSI scanning on three occasions at both 1.5T and 3T, with four scans acquired at each visit. We measured variance components, correcting for grey matter and white matter content of voxels, of metabolite peak areas and peak area ratios.

**Results:**

Residual variance was in general the largest component at 1.5T (8.6–24.6%), while within-brain variation was the largest component at 3T (12.0–24.7%). Inter-subject variation was around 5%, while inter- and intra-session variance were both generally small.

**Conclusion:**

Multiple variance contributions associated with MRSI measurements were quantified and the performance of 1.5T and 3T MRI scanners compared using data from the same group of subjects. Residual error is much lower at 3T, but other variance components remain important.

## Introduction

Exploitation of the ^1^H chemical shift effect permits quantitative mapping of metabolite signals across the brain and within other organs of the body. Single voxel spectroscopy and magnetic resonance spectroscopic imaging (MRSI) may be used diagnostically and for assessing treatment effects in medicine and clinical research, particularly in oncology [1]. MRSI sequences are now widely available as standard on 1.5T and 3T clinical MRI scanners. Although a number of test-retest reliability studies have been published [2–11], most are based on small sample sizes or focus on a limited number of variance components; furthermore, while 3T MRI is often regarded as essential for clinical research involving MRS, there is little comparative information available regarding reproducibility at 1.5T versus 3T [6, 12].

The aim of the present study was to measure five key variance components of MRSI. While intra-session variance (i.e. scan-to-scan, including short-term scanner instability, and limitations in the shimming and other adjustment procedures) is important for measuring metabolite levels cross-sectionally and for measuring within-session changes, it is also important to characterise inter-session variability (i.e. day-to-day variation, including that due to biological changes and day-to-day scanner instability) where longitudinal changes are to be measured over a longer period. We also measured inter-subject, within-brain (to account for variance due to anatomy, coil sensitivity inhomogeneity etc.) and residual (e.g. variability due to scanner noise) variances.

## Materials and methods

### Study design

To estimate the variance components, we scanned a group of healthy volunteers, performing repeat scans both within each session and on multiple days, analysing the resulting metabolite peak areas and their ratios using linear mixed effects models. In addition, we used structural imaging to correct for the different proportions of grey matter (GM) and white matter (WM) in the MRSI voxels. The same volunteers were scanned at both 1.5T and 3T magnetic field strengths. All procedures performed in the study were conducted in accordance with the principles expressed in the Declaration of Helsinki and following approval by the regional ethics committee. Written informed consent was obtained from all individual participants included in the study. We recruited 11 subjects (age range 23–40, mean 30.5 years), who were invited for scanning on three occasions in both 1.5T and 3T scanners; all six scanning visits took place in the afternoon with no more than 2 months elapsing between the first and last visits; four MRSI scans were acquired consecutively during each session. Since the primary purpose of obtaining the data was to evaluate MRSI for brain temperature measurement [13], only male subjects were recruited in order to avoid the possible confound of temperature variation during the menstrual cycle.

### Magnetic resonance imaging

**Fig 1:**
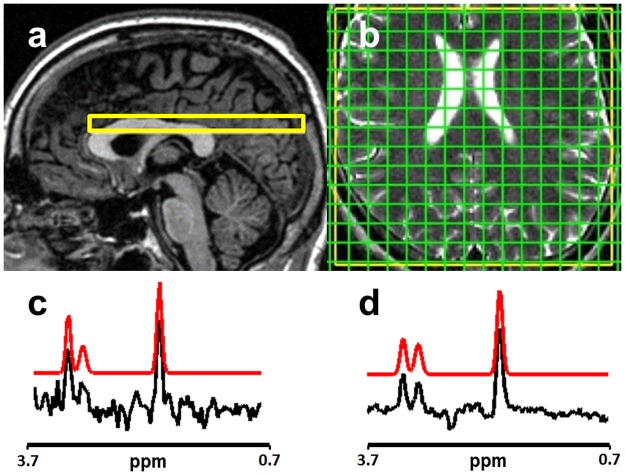
MRSI slice location and spectra. 1.5T MRSI volume of interest (yellow) overlaid on (a) sagittal localiser image and (b) axial T2W image. (c) and (d) show typical MRSI spectra acquired at 1.5T and 3T respectively with fitted spectra overlaid in red. The chemical shift is displayed relative to the water resonance frequency.

All 1.5T scanning was performed using a GE Signa Horizon HDxt clinical MRI scanner (GE Healthcare, Slough, UK) fitted with a transmit-receive quadrature head coil. Spectroscopy data were obtained using the manufacturer-provided PRESS MRSI pulse sequence (TR/TE = 1000/144 ms, field of view (FOV) = 300×300 mm) with 24-step phase encoding for both in-plane MRSI grid axes. A single 10 mm slice was located axially at the level of the superior part of the corpus callosum using axial T2-weighted (T2W) and sagittal localiser images for planning, as shown in Fig 1a and 1b. Four saturation bands were applied to suppress scalp lipid signals and the excitation region was restricted to the anterior extent of the corpus callosum to minimise signal from regions with poor magnetic field homogeneity. Automated shimming and CHESS water suppression, which was adjusted to retain a residual water signal, were applied. For each phase encoding step, a 512 ms free induction decay (FID) was obtained with 1 ms dwell time. The MRSI scanning time was approximately 9 m 40 s following prescan optimisation, which was performed before every acquisition. Localiser images and axial T2-weighted scans (two-dimensional fast spin-echo (FSE) sequence, TR/TE = 11320/102 ms, matrix = 256 × 256, FOV = 256 × 256 mm, 2 mm thickness contiguous slices) were acquired prior to MRSI to facilitate placement of the volume of interest. Additional T1-weighted (T1W; 3D inversion-recovery-prepared spoiled gradient echo (GE), TR/TI/TE = 9.6/500/4.0 ms, FA = 8°, 1.3 mm isotropic resolution) and axial 2D GE (TR/TE = 940/15 ms, FA = 20°, matrix = 256 × 192, FOV = 256 × 256 mm, 2 mm thickness contiguous slices) scans were acquired at the first visit only.

3T scanning was performed using a Magnetom Verio 3T clinical MRI scanner (Siemens, Healthcare Gmbh, Erlangen, Germany) equipped with a 12-channel receive-only head coil. MRSI data were obtained as described above using a manufacturer-provided semi-LASER PRESS MRSI sequence [14] (TR/TE = 1700/144 ms, FOV = 300×300 mm); elliptical *k*-space sampling was used to achieve a similar scan time (approximately 10 m 50 s) to the 1.5T acquisition; data from the coil elements were phase corrected individually and combined in the time domain using the manufacturer-provided algorithm [15]. Axial T2W (2D FSE, TR/TE = 13241/98 ms, matrix = 256 × 256, FOV = 256 × 256 mm, 2 mm thickness contiguous slices), T1W (3D inversion-recovery-prepared spoiled GE; TR/TI/TE = 2300/900/2.98 ms, FA = 9°, 1 mm isotropic resolution) and axial 3D GE (TR/TE = 27/10 ms, FA = 15°, matrix = 256 × 256, FOV = 256 × 256 mm, 2 mm slice thickness) scans were also obtained at the first visit.

### Data analysis

MRSI data, interpolated to 32 × 32 voxels (nominal voxel dimensions 9.375 × 9.375 × 10 mm) by zero-filling of the k-space data, were corrected for phase and eddy current distortion using the residual water signal [16]; N-acetyl aspartate (NAA), creatine (Cre) and choline (Cho) metabolite time-domain signal amplitudes (proportional to peak area in the frequency domain and henceforth referred to as “peak area”) were determined by fitting the data to a Gaussian lineshape model using the AMARES algorithm [17] following removal of the water resonance (HLSVD algorithm; [18]) in the JMRUI software package [19]. Peak areas were corrected for any changes in receiver and transmitter gains based on information provided by the manufacturers (for 1.5 T MRSI, amplitudes were scaled by $2^{\frac{1-R1}{2}+30-R2}\times 10^{\frac{TG-65}{200}}$, where *R1*, *R2* and *TG* are the receiver and transmitter gains recorded prior to the start of each MRSI scan; for 3T, amplitudes were multiplied by the transmitter reference voltage stored in the header of the MRSI raw data file), yielding values in “institutional units” (IU). Finally, NAA/Cre, NAA/Cho and Cho/Cre signal amplitude ratios were calculated.

T1W and GRE images obtained at the first visit were co-registered to T2W scans using FSL FLIRT [20]; brain masks were derived from GRE images using FSL BET2 [21] and applied to T1W images, from which tissue segmentation images were generated using FSL FAST [22]. Segmentation was performed separately using structural scans acquired at the two field strengths and the quality was checked visually. Voxels outside the volume of excitation, or covering regions containing > 5% CSF or > 5% non-brain-tissue volume were excluded from the analysis. Finally, all spectra were reviewed by an experienced MR physicist and discarded if they had (i) severely distorted baselines (e.g. due to poor water suppression or lipid contamination) and/or (ii) poor shimming or excessive noise that made the choline and creatine peaks difficult to resolve.

### Statistics

Metabolite peak area and peak area ratio data were analysed using a linear mixed effects model (*fitlme* function in Matlab (Mathworks, Natick, MA)). The models included the random effects of subject number, visit number, scan number and voxel number, which were reported as standard deviations; to better facilitate comparison between scanners and metabolites, these were plotted as percentage coefficients of variation (CV), normalised using the mean of all measurements. For signal peak area, the model was corrected for the covariates $\Delta^{IU}=\frac{1}{2}(f_{GM}-f_{WM})$ and Σ = *f*_GM_ + *f*_WM_, to account for the effects of GM-WM differences and total brain tissue content of voxels respectively; *f*_GM_ and *f*_WM_ are the volume fractions of grey matter and white matter respectively. The random effects were nested according to the model equation:

$${signal}_{ijkl}=\beta_{0}+{subject}_{i}+{visit}_{ij}+{scan}_{ijk}+{voxel}_{ijl}+\Delta_{ijl}^{\text{IU}}+\Sigma_{ijl}+\varepsilon_{ijkl}.$$

Note that the voxel effect was not nested within scan, as it was assumed that subjects did not move between scans within a session. Since the metabolite signal ratios depend only on the relative amounts of GM and WM, the model included a single covariate:

$${ratio}_{ijkl}=\beta_{0}+{subject}_{i}+{visit}_{ij}+{scan}_{ijk}+{voxel}_{ijl}+\Delta_{ijl}^{\text{ratio}}+\varepsilon_{ijkl},$$

where *$\Delta^{ratio}=\frac{1}{2}\left(f_{GM}-f_{WM}\right)/\left(f_{GM}+f_{WM}\right)$. P* < 0.05 (two-sided) was regarded as significant in statistical tests. Voxel-by-voxel data for spectra located within the excitation volume is supplied as S1 Table.

## Results

We obtained MRSI data at all scanning visits. One subject withdrew from the study following a single 1.5T visit; a second subject attended three 3T visits but only one 1.5T visit; a single 1.5T MRSI scan was excluded from the analysis due to failure of the scanner optimisation procedure; finally, peak areas obtained at a single 1.5T visit were discarded as outliers, having approximately twice the amplitude compared with other visits and subjects for all three metabolites; this was likely caused by an error in the setup or the optimisation procedure. Of the total number of “brain voxels” (defined as those covering at least 95% GM, WM or CSF) acquired, 52% and 51% of these contained >5% CSF and were excluded from the analysis at 1.5T and 3T respectively; 36% and 11% of the remaining voxels were excluded based on visual assessment of spectral quality (e.g. due to poor water suppression, lipid contamination or peaks not clearly visible due to poor field homogeneity); in total, 31% and 43% of all brain voxels were retained for statistical analysis.

### Linear mixed effects model

**Fig 2:**
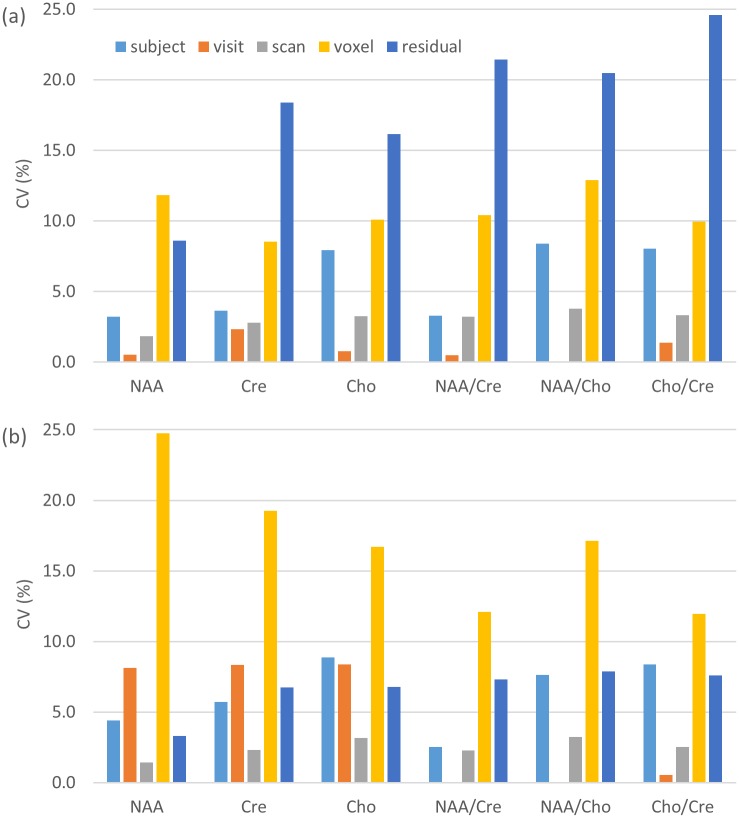
Metabolite signal variance components. Variance components of the metabolite signal peak areas and peak area ratios at 1.5T (a) and 3T (b). To facilitate comparison between scanners and metabolites, the values shown are percentage CVs.

MRSI data were analysed separately for each scanner using a linear mixed effects model, including covariates to account for tissue class content, as described in the Materials and Methods section. The results, shown in Table 1 and Fig 2, demonstrate that inter-subject effects were similar across the scanners and quantities measured (CV = 2.5–8.9%). The variance due to the effect of visit (i.e. inter-session) was small (CV ≤ 2.3%) at 1.5T and for peak area ratio measurements at both field strengths, but considerably larger (CV = 8.1–8.4%) for peak area measurements at 3T. The variance associated with repeat scanning (i.e. intra-session) was small (CV ≤ 3.8%) for all measurements. The effect of voxel (i.e. within-brain variance, corrected for GM/WM content) was in the range CV = 8.5–24.7% and was the largest variance component at 3T. The residual variance (i.e. that remaining once the effects of subject, visit, scan and voxel have been accounted for) was in general the largest variance component at 1.5T (CV = 8.6–24.6%) and had approximately half the magnitude at 3T (CV = 3.3–7.9%); the residual was smaller for peak areas than for peak area ratios.

**Table 1:** Mean metabolite signal values and variance components, estimated using a linear mixed effects model.

	metabolite peak area (IU)			metabolite peak area ratio		
	NAA	Cre	Cho	NAA/Cre	NAA/Cho	Cho/Cre
**1.5 T MRI**						
**mean**	121	54	65	2.3	1.9	1.3
**σ**_**subject**_	3.9*	1.9	5.2*	0.08	0.16*	0.10*
**σ**_**visit**_	0.6	1.2	0.5	0.01	0.00	0.02
**σ**_**scan**_	2.2*	1.5*	2.1*	0.08*	0.07*	0.04*
**σ**_**voxel**_	14.3*	4.6*	6.6*	0.24*	0.25*	0.13*
**σ**_**residual**_	10.4*	9.8*	10.5*	0.50*	0.40*	0.31*
**β**_**Δ**_	-13.3*	8.1*	-20.7*	-0.6*	0.5*	-0.6*
**β**_**Σ**_	16.6	13.5	31.7	—	—	—
**3.0 T MRI**						
**mean**	855	378	333	2.3	2.6	0.9
**σ**_**subject**_	37.5	21.5	29.5	0.06	0.20*	0.08*
**σ**_**visit**_	69.3*	31.5*	27.9*	0.00	0.00	0.00
**σ**_**scan**_	12.1*	8.7*	10.6*	0.05*	0.08*	0.02*
**σ**_**voxel**_	211.5*	72.7*	55.7*	0.27*	0.44*	0.11*
**σ**_**residual**_	28.3*	25.5*	22.6*	0.16*	0.20*	0.07*
**β**_**Δ**_	-181.4*	9.3	-81.8*	-0.5*	0.2*	-0.3*
**β**_**Σ**_	35.2	144.5	231.0*	—	—	—

The mean values for each quantity, averaged over all accepted voxels, are also shown. Note that the covariate Δ equals $\frac{1}{2}(f_{GM}-f_{WM})$ for metabolite peak areas and $\frac{1}{2}\left(f_{GM}-f_{WM}\right)/\left(f_{GM}+f_{WM}\right)$ for peak area ratios (“*” indicates *P* < 0.05).

Inspection of the β_Δ_ coefficients (Table 1) shows that both NAA and Cho peak areas fell with increasing GM content at both 1.5T and 3T, while Cre peak areas increased with increasing GM content (*P* < 0.05 at 1.5 T only). NAA/Cre and Cho/Cre peak area ratios decreased with increasing GM as a proportion of combined GM and WM, while the opposite trend for NAA/Cho was observed at both field strengths.

## Discussion

The aim of our study was to determine the variance components at 1.5T and 3T for MRSI measurements of metabolite peak areas and peak area ratios. In general, within-brain variation and the residual variance due to the noise were the largest variance components. The residual variance was the dominant variance component at 1.5T. Since this probably reflects the influence of the signal-to-noise ratio in addition to other factors not explicitly modelled, it is not surprising that it was approximately threefold lower at 3T, where the combined benefits of a higher magnetic field strength and a 12-channel phased-array coil (compared with a transmit-receive quadrature coil at 1.5T) result in higher signal-to-noise ratio. The residual variance was somewhat greater for peak area ratios than for peak areas, which is expected since the former are subject to the error in two measurements; previous studies have reported similar differences in findings for peak areas and peak area ratios [2, 8].

Within-brain variation, irrespective of voxel GM and WM content, was the second largest variance component at 1.5T. At 3T, within-brain variation was the largest variance component for all quantities measured, consistent with earlier studies of short-echo-time MRSI [4, 9]. Within-brain variation at 3T was similar to that measured at 1.5T for peak area ratios but was larger for peak areas. This may result from greater radiofrequency transmit and receive inhomogeneity at 3T, as well as differences in chemical shift displacement of the excitation volume and the pulse excitation and refocussing profiles; these effects should be reduced for metabolite signal ratios; the higher proportion of retained voxels at 3T (43% versus 31%) could also contribute. Examination of the coefficients for the predictors used to account for GM and WM content suggest that NAA and Cho peak areas, and NAA/Cre and Cho/Cre ratios were significantly higher in voxels containing less GM content (i.e. absolute GM content for peak areas or GM content as a fraction of total GM and WM tissue for peak area ratios), while the NAA/Cho ratio was significantly lower. These trends were similar at 1.5T and 3T, and consistent with the findings of a study of elderly volunteers using a similar protocol at 1.5T with the imaging plane located at the level of the basal ganglia [23]; however, literature reports vary in this regard (e.g. [24, 25] and references cited in [23]), as do the acquisition methods, quantification methods and anatomical locations used.

Both inter- and intra-session variance components were small, suggesting that temporal variation in physiology and scanner performance are not major influences (it should be noted that inter-session components represents the *additional* variance for scans obtained on different versus the same days, and not the overall variance). Jackson et al. also found variation between acquisitions to be less important than within-brain or inter-subject variation [7]; two other studies also found little difference between intra- and inter-session reproducibility [5, 10]. Nevertheless, inter-visit variation was noticeably larger for peak areas measured at 3T. We suggest this may be due to our use of “institutional units”, which were corrected for coil loading using the transmitter gain (or transmit reference voltage at 3T) and receiver gain settings, although the variability in the correction factor was similar for the two scanners (CV: 6.0 and 4.6% for scans at 1.5 T and 3 T respectively). This correction yields stable (in the short-medium term) peak areas at 1.5T, where a transmit-receive coil was used. However, when a receive-only head coil is used, as here at 3T, changes in the ratio between receive and transmit coil sensitivities (e.g. due to differences in patient positioning) may cause additional variance unless further calibration scans are performed [26]. This should not affect the peak area ratios, for which the inter-session variance contribution was minimal using both scanners. A number of alternative methods for calculating institutional units and for performing absolute quantification are available [27]. For example, the subject can be replaced with a solution of known concentration which is scanned with identical gain settings; such “phantom replacement” approaches allow estimates of absolute concentration provided the phantom loads the coils in the same way as the subject. Alternatively, metabolite peak areas can be expressed relative to the water peak area, thereby reducing scanner-dependent influences. However, the increase in overall acquisition time, which may be acceptable for single voxel MRS, is often prohibitive for MRSI; interleaved acquisition of the water signal is possible using echo planar spectroscopic imaging [28], although this method is not widely available clinically at present. As a minimum, regular quality assurance scanning of a phantom should be performed to gauge the temporal stability of institutional units.

Inter-subject variance was in the range 2.5%– 8.9%, with the choline peak area and ratios involving choline at the higher end of this range. Reassuringly, the inter-subject variance was similar at both magnetic field strengths.

A strength of the study is that we obtained a large amount of data (236 MRSI scans) compared with most previous test-retest studies, comprising multiple scans on multiple occasions in a reasonable number of subjects, which allowed us to disentangle the variance components associated with MRSI metabolite measurements. In addition, subjects were scanned using both 1.5T and 3T scanners. A weakness of the study is that we did not perform MRSI at short TE for measurement of lower-amplitude metabolites such as myo-inositol and glutamate. In addition, it should be noted that differences in scanner generation, software and hardware (notably differences in the receive coils) as well as magnetic field strength will affect the findings, limiting our ability to make a pure comparison between the two field strengths; however, while our 3T data is likely more representative of reliability in a modern clinical research setting, it is interesting to note that some of the variance contributions, e.g. inter-session and inter-voxel, were often higher for the 3T scanner. Finally, we performed two-dimensional MRSI covering a single slice in the brain, since this is the most widely used method clinically. However, more recently developed echo-planar spectroscopic imaging approaches permit whole-brain MRSI to be acquired in a similar scan time, facilitating intra-subject co-registration and inter-subject normalisation, allowing variance to be estimated as a function of anatomical location (e.g. [3, 10]).

## Conclusions

In conclusion, we have measured the variance components associated with metabolite signal peak areas and peak area ratios at 1.5T and 3T MRSI. Our data indicate that, at 1.5T, residual error likely due to scanner noise was the dominant variance component, followed by within-brain variation. At 3T, the residual error was much lower, with within-brain variation the largest single component. Our findings confirm the expected benefit of increased field strength coupled with high sensitivity coils, though other components, such as within-brain and inter-session variance, remain important at 3T. Inter-subject variance was approximately 5% for healthy participants at both field strengths.

## Supporting information

S1 Table Voxel-by-voxel data. The data fields are described in the second row of the table.
(CSV)
